# Supplementary figures and images for: Out of Africa by spontaneous migration waves
Source: PLoS One. 2019 Apr 23;14(4):e0201998. doi: 10.1371/journal.pone.0201998 (PMC6478371; doi:10.1371/journal.pone.0201998)

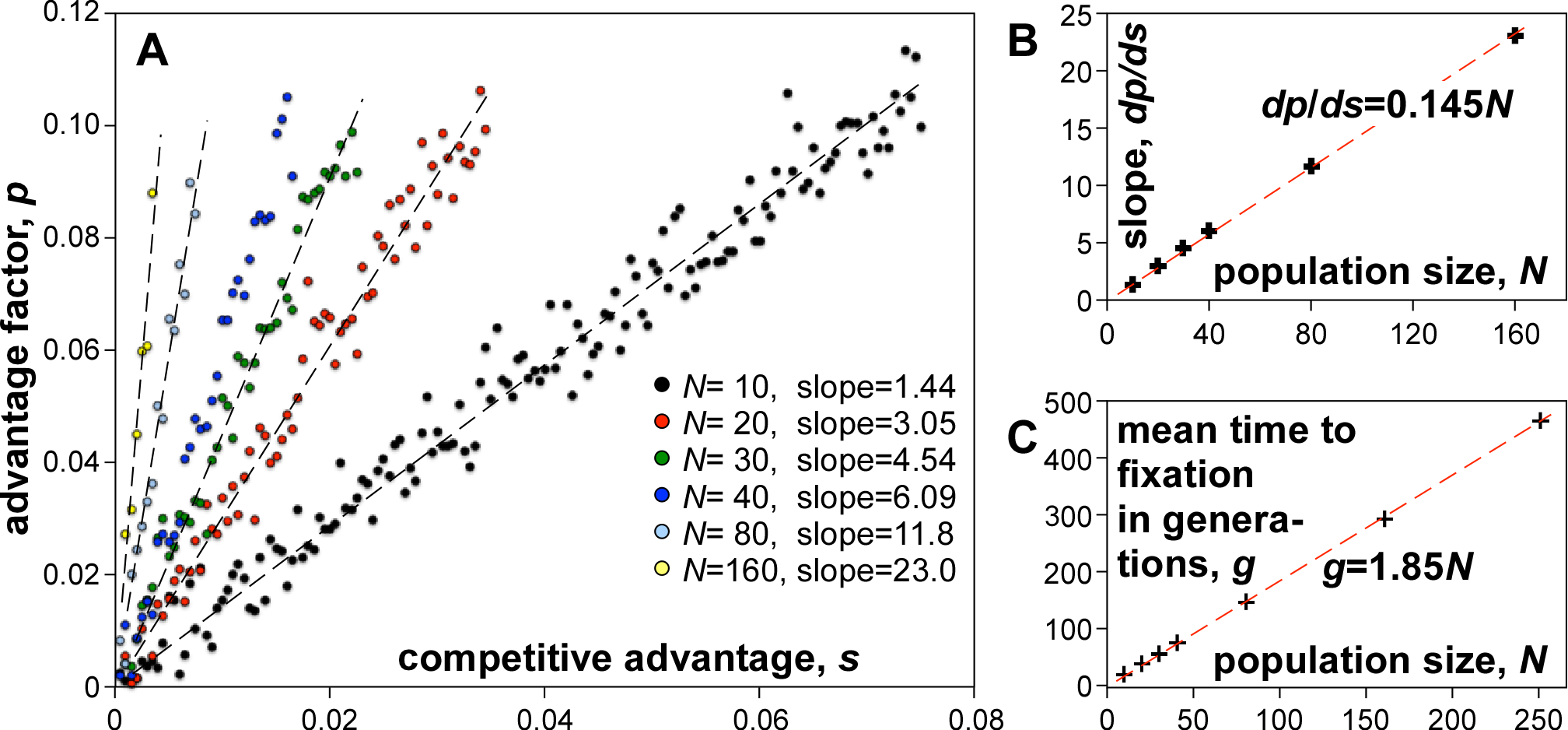

Supplement: S1 Fig — A. The factor p as a function of competitive advantage s and population size N. Each data point is represents the average 10,000 simulations for a give N and s. Slopes (dP/ds) are determined by linear least-squares best fits (dashed lines). B. A regression (dashed line) shows that dP/ds is a linear function of N. C. Modelled mean time to fixation of a neutral mutation is found to be a linear function of N. (TIF) [file pone.0201998.s001.tif]

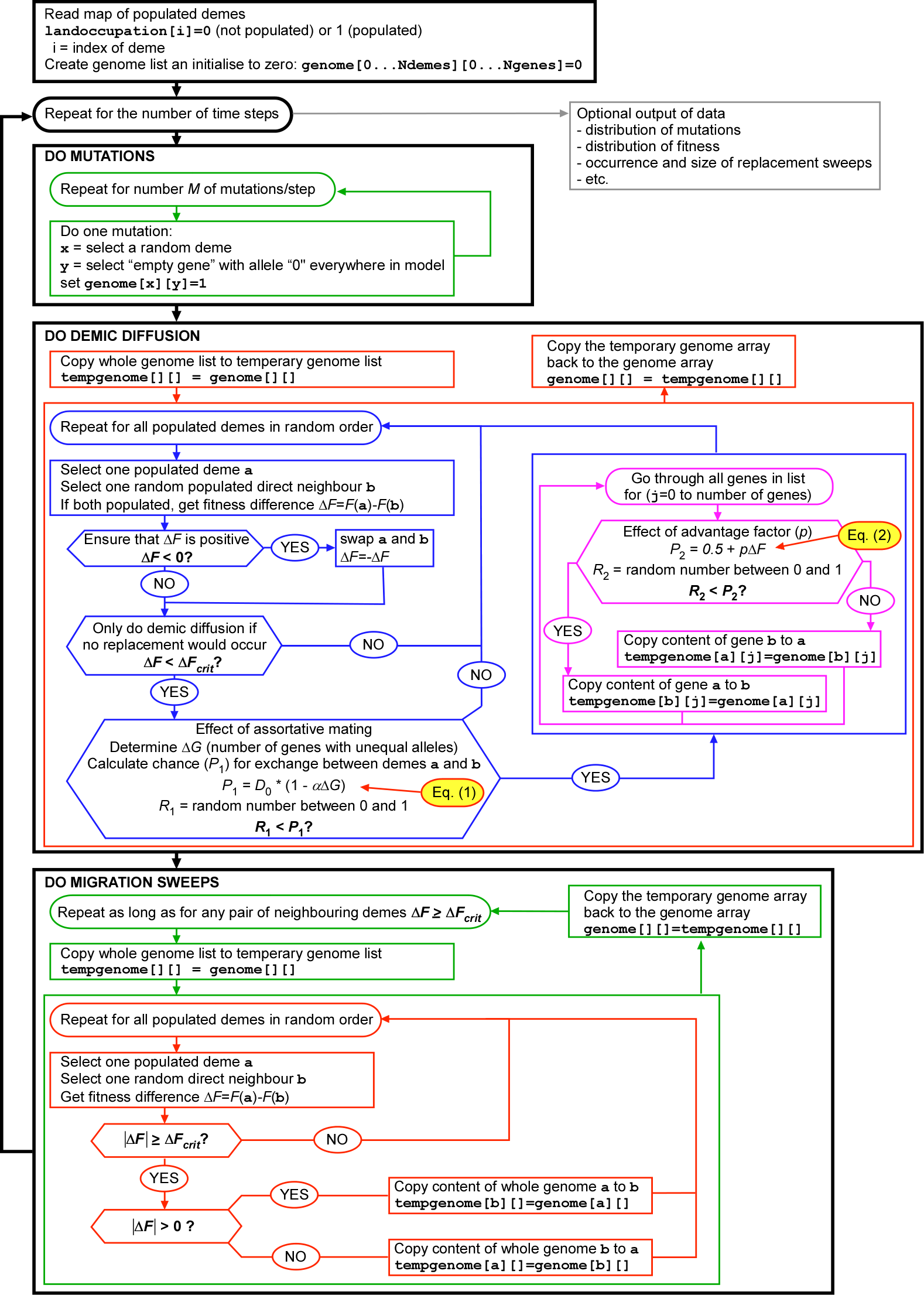

Supplement: S2 Fig — Individual loops are given in different colours. (TIF) [file pone.0201998.s002.tif]
