# Supplementary material for: Out of Africa by spontaneous migration waves
Source: PLoS One. 2019 Apr 23;14(4):e0201998. doi: 10.1371/journal.pone.0201998 (PMC6478371; doi:10.1371/journal.pone.0201998)
Supplement: S1 File — The text explains the relationship between advantage factor (p) and competitive advantage (s) and how the duration of one time step (Δt) is derived. (PDF) [file pone.0201998.s003.pdf]

Out of Africa by spontaneous migration waves

PLOS ONE, 2019

Paul D. Bons, Catherine C. Bauer, Hervé Bocherens, Tamara de Riese, Dorothée G. Drucker, Michael Francken, Lumila Menéndez, Alexandra Uhl, Boudewijn P. van Milligen, Christoph Wißing

Supporting information on methods

Relationship between advantage factor (*p*) and competitive advantage (*s*)

To determine what the time step (Δt) in the model is, we consider the exchange between two neighbouring demes, one carrying a mutation with a competitive advantage *s*. The chance that this mutation is copied to the other deme in one time step (if D_0=1) is 0.5+*p* (Eq. 3). We thus need to find the time it takes for a competitive mutation carried by 50% (f_0=0.5) of a population of size $2N_D$ (N_D is the population of one deme). A range of models exists to predict the chance of and the time to fixation [58-61,63,108] for a range of cases (diploid or not, recessive or dominant, etc.). Equations for our purpose here are, however, not readily available. We therefore use a simple numerical model, to estimate Δt for a given *p* and N_D.

The model has *N* individuals, each carrying either 0, 1 or 2 copies of the mutation. Each generation, each individual mates with two randomly selected other individuals, each time producing one offspring that carries one randomly selected allele from the two parents. If the two alleles contain one or two copies of the mutation, another offspring is created with a chance of *s*/2 or *s*, respectively. The number of offspring in the next generation is thus slightly larger than *N*, because of the additional offspring stemming from the parents that carry the competitive mutation. To keep the population constant, *N* individuals are randomly selected from the offspring. This is repeated until the mutation has reached fixation or has completely disappeared from the population.

The fixation probability, i.e. the total number of fixation events divided by number of simulations (here 10,000x) equals 0.5+*p*. For small values of *s*, $p_{(N)}$ increases linearly with *s*, with a slope *a* that depends on *N* (S1 Fig.). The slope *a* is itself linearly proportional with *N* (S1 Fig.), resulting in a linear relationship between $p_{(N)}$ and *s*:

$$p_{(N)} = 0.145sN\,. \qquad \text{(A)}$$

The population N_D of a deme is the product of the population density ρ and size *S* of the deme, giving (using $N=2N_D$):

$$p = 0.29s\rho S^2 \quad \text{or} \quad s = \frac{p}{0.29\rho S^2}\,. \qquad \text{(B)}$$

Duration of one time step (Δt)

When starting with 50% of the population with a mutation, the number (T) of generations (of g years/generation) to reach fixation or extinction of the mutation decreases slowly with s, when s is small. For $s \approx 0$ we can therefore approximate $T_{(s)}$ with $T_{(s=0)}$. A plot of mean $T_{(s=0)}$ as a function of N shows a linear relationship with a slope of 1.85 generations/individual (S1 Fig.). Considering that an exchange between two demes is, on average, only considered $1/D_0$ times per two time steps and $N=2N_D$, we get for the duration of one time step (Δt):

$$T \approx 1.85N = 7.4\frac{\rho S^2}{D_0} \Leftrightarrow \Delta t = 7.4\frac{\rho S^2 g}{D_0}. \qquad \text{(C)}$$

We see that the time step depends on the size S of the demes, but not on the competitive advantage (s). Changing the size of demes (while keeping all other parameters the same) should not affect the velocity (in km/yr). Spreading velocity of a deme depends on its competitive advantage s, and hence on p (Eq. B). It should not depend on deme size S. Simulations show that the velocity in demes per time step is approximately proportional to the square root of p (Eq. 7).

$$v\left[demes/timestep\right] \propto p^{1/2} \Leftrightarrow v\left[km/yr\right] \propto \frac{p^{1/2}S}{\Delta t} = \frac{(s\rho)^{1/2}S^2}{\rho S^2 g} = \frac{s^{1/2}}{\rho^{1/2}g} \qquad \text{(D)}$$

This shows that the spreading velocity is indeed approximately independent of model resolution, but that p should be chosen depending on s, population density and resolution. The slight dependency on model resolution (the exponent in Eq. 7 is not 0.5, but 0.54) is explained by the resolution of the diffusional front.

In our simulations presented here, we mostly use p=0.05 and S=50 km as a compromise between resolution and computational effort. If we assume a population density of between 0.01 and 0.1 individuals/km^2, the choice of p and S implies that the competitive advantage of the mutations is between 0.07% and 0.7% (Eq. B). The resulting time step, assuming g=25 years/generation, is between about one and ten thousand years (Eq. C).

References

58. Whitlock MC. Fixation Probability and Time in Subdivided Populations. Genetics. 2003; 164: 767–779.
59. Fisher RA. The Genetical Theory of Natural Selection. Oxford: Oxford University Press; 1930.
60. Fisher RA. The wave of advance of advantageous genes. Ann Eugen. 1937; 7: 355-369. doi: 10.1111/j.1469-1809.1937.tb02153.x.
61. Kimura O, Ohta T. The average number of generations until fixation of a mutant gene in a finite population. Genetics. 1969; 61: 763-771.
63. Haldane JBS. A mathematical theory of natural and artificial selection. V Proc Camb Philos Soc. 1927; 23:838 – 844.
108. Patwa Z, Wahl LM. The fixation probability of beneficial mutations. J. R. Soc. Interface. 2008; 5: 1279–1289. doi:10.1098/rsif.2008.0248.
